# Functional Predictors for Home Discharge after Hip Fracture in Patients Living in Sloped Neighborhoods or Islands: An 8-Year Retrospective Cohort Study

**DOI:** 10.3390/geriatrics5040093

**Published:** 2020-11-15

**Authors:** Yuta Suzuki, Noriaki Maeda, Naoki Ishibashi, Hiroaki Murakami, Masanori Morikawa, Junpei Sasadai, Taizan Shirakawa, Yukio Urabe

**Affiliations:** 1 Department of Sports Rehabilitation, Graduate School of Biomedical and Health Sciences, Hiroshima University, Hiroshima 734-8553, Japan; yt.suzuki28@gmail.com (Y.S.); norimmi@hiroshima-u.ac.jp (N.M.); m-masanori@hiroshima-u.ac.jp (M.M.); 2 Department of Rehabilitation, Matterhorn Rehabilitation Hospital, Hiroshima 737-0046, Japan; rugby1216nn@gmail.com (N.I.); bm-vaaaaa@outlook.jp (H.M.); 3 Sports Medical Center, Japan Institute of Sports Sciences, Tokyo 115-0056, Japan; jumpei.sasadai@jpnsport.go.jp; 4 Department of Orthopedics, Matterhorn Rehabilitation Hospital, Hiroshima 737-0046, Japan; matter@jasmine.ocn.ne.jp

**Keywords:** hip fracture, elderly, discharge destination, neighborhood, slope, island

## Abstract

Functional predictors of home discharge after hip fractures have been widely reported; however, no study has considered the geographical features surrounding patients’ homes. This study aimed to identify home discharge predictors and determine the cutoff points required for home discharge of patients living in sloped neighborhoods or islands. A total of 437 postoperative hip fracture patients were included and classified into the flat, slope, and island groups according to their residential area before the fracture. Multivariate logistic regression analysis was used to identify significant home discharge predictors, and receiver-operating characteristic analysis to calculate cutoff values. In all the groups, the functional independence measure-motor score was a significant home discharge predictor, with cutoff values of 69 for the flat group and 65 points for the slope and island group. In the slope group, the 6-minute walking distance (odds ratio, 1.02; 95% confidence interval, 1.01–1.04) and revised Hasegawa dementia scale score (odds ratio, 1.06; 95% confidence interval, 1.01–1.12) were also identified as predictors, with cutoff values of 150 m and 18 points, respectively. The outcomes required for home discharge after hip fracture differ depending on the neighborhood terrain, especially for patients living in areas with many slopes and stairs.

## 1. Introduction

Hip fracture is the most common injury that threatens life prognosis in the elderly. Its incidence rate has continued to increase because of the greater population aging in Japan, and the number of new patients with hip fracture in 2012 was estimated to be 175,700 [1]. The final goal of rehabilitation for patients after hip fracture is to return to their own home and regain their pre-fracture level of activity. Several previous studies highlighted the need to restore walking ability and functional status at an early stage by rehabilitation to extend the healthy life expectancy and quality of life of the elderly [2,3]. However, 40–57% of patients had improved mobility to the same level as before surgery for hip fracture and 13% were still unable to walk 1 year after surgery [4,5]. In 40–70% of patients, the pre-fracture level of independence in basic activities of daily living (ADL) was regained [6]. Therefore, many patients fail to achieve the rehabilitation goal of discharge to home after hip fracture because they could not recover their pre-fracture level of mobility and ADL ability.

Kure City has the highest aging rate of 35%, with a population of >150,000 in 2019 in Japan. Furthermore, the city has the third largest proportion of urban slopes and stairs in Japan because it is surrounded by multiple mountains [7] and has five inhabited islands, as Japan is bordered by the ocean. Thus, many elderly people live in sloped residential areas and islands. Sloped terrain may act as an environmental barrier for walking [8]. In an island, walking independence is a determinant of the frequency of going out [9], and elderly people with reduced mobility and ADL ability will not be able to continue living in the island [10]. These studies suggest that such geographical features around homes make it more difficult for the elderly to be discharged to their homes, especially if they have not regained their pre-fracture level of mobility and ADL independence.

Postoperative treatment should be planned for patients to recover from their impaired physical functional status and return to their own home environment. Numerous studies have already assessed predictors of home discharge after hip fracture, including age, sex, walking independence, cognitive function, level of independence in ADL, and family structure [11,12,13,14]. However, for patients after hip fracture, no report has considered the geographical features surrounding patients’ homes. Given that there are many sloped urban cities and inhabited islands in the world, including Japan, the predictors of discharge destination after hip fracture in patients living in sloped residential areas and islands may provide important knowledge to establish treatment and rehabilitation after hip fracture. Moreover, the values of specific criteria related to the home discharge of patients living in those terrains may be important indicators for health care providers, including medical doctors, to decide where to discharge.

The first objective of this study was to identify cognitive and physical predictors of discharge to home for patients living in sloped terrains or islands after a hip fracture. The second objective was to determine the cutoff point of the functional score that best differentiates patient discharge to home in sloped terrains or islands.

## 2. Materials and Methods

### 2.1. Study Design and Subjects

**Figure 1:**
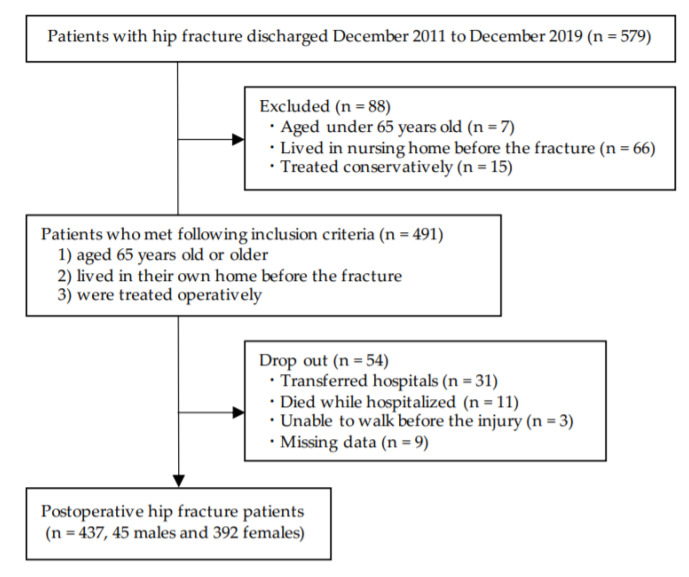
Patient selection process.

This study was conducted as a retrospective cohort study. Participants were identified from the patient database of a convalescent rehabilitation hospital with a 94-bed capacity in Kure City, Hiroshima Prefecture, Japan. This study included all consecutive 579 patients with hip fractures who were discharged between December 2011 and December 2019 (a period of 8 years). All 579 patients were admitted to our convalescent unit in the hospital from an acute care hospital and reeducated at our rehabilitation institution. The inclusion/exclusion criteria and selection process are shown in Figure 1. Finally, 437 postoperative hip fracture patients (45 men and 392 women) were included in this study. All the patients received a standard physical therapy (i.e., range of motion, muscular strength, walking, climbing stairs, and balance exercise) and occupational therapy (i.e., practice of ADL) for 3 h a day, 6 or 7 days a week.

This study was reviewed and approved by the institutional review board of Matterhorn Rehabilitation Hospital (approval ID: MRH19007, date: 17 December 2019).

### 2.2. Demographics of the Participants and Group Definition

Demographic characteristics were collected from medical records, including age, sex, height, weight, body mass index (BMI), type of fracture operative procedure, place of residence before the fracture (flatter residential area [flat group], sloped residential area [slope group], or isolated island [island group]), family structure, discharge destination, and period of hospitalization.

To identify the place of residence before the fracture, we first reviewed the living conditions before the injury obtained from the medical questionnaire at admission by the patients or their families, or the conference records for discharge assistance, and then examined the neighborhood terrain around the home based on the patient’s address. Finally, the neighborhood terrain around the homes of all the subjects was checked using Google Maps or the maps provided by the Geospatial Information Authority of Japan, and if there was any doubt about the decision, we went to the site to confirm it. Patients living in sloped residential areas (slope group) were defined as those who required walking on slopes or stairs over at least 200 m [15] when going out in daily life because of the geographical features surrounding their homes. Patients who were living in the five inhabited islands before the fracture were assigned to the island group.

The discharge destination was determined in a conference attended by patients, families or key persons, medical doctors, nurses, rehabilitation staffs, and social workers, with considering the patient’s physical and cognitive functions, ADL ability, and expected living conditions after discharge.

### 2.3. Functional Outcomes

All the patients underwent the following assessments of six functions as predictors of discharge to home 4 to 5 weeks after their admission: cognitive function (revised Hasegawa dementia scale, HDS-R), grip strength, walking speed (10-m walking test, 10mWT), walking endurance (6-min walking distance, 6MWD), balance ability (one-leg standing time, OLST), and ADL ability (functional independence measure, FIM). HDS-R can screen for cognitive impairment accurately and efficiently and generally reflects dementia, including mild cognitive impairment [16,17]. The FIM is an 18-item measurement tool that has two subunits. The motor subunit is comprised of 13 items that relate to self-care, sphincter control, transfer, and locomotion, and the cognitive subunit is comprised of five items that evaluate comprehension, expression, and memory [18]. Each item is rated with a grade of 1–7 points ranging from “total assistance with a helper” to “complete independence.”

### 2.4. Statistical Analyses

Data analysis was performed using JMP Pro version 14.2 for Mac (SAS Institute Inc., USA). Demographic characteristics were compared among the residential areas using one-way analysis of variance for quantitative variables and the chi-square test for qualitative variables. When appropriate, a follow-up analysis was performed using the Tukey–Kramer test for quantitative variables. Furthermore, a two-way ANOVA (3 [group] × 2 [discharge destination]) was conducted for six predictors of discharge to home: cognitive function (HDS-R), physical function (grip strength, 10mWT, 6MWD, and OLST), and ADL ability (FIM). When interaction effects were detected, post hoc comparisons were performed to test the differences in variables.

A univariate analysis for each of the six functional assessments was conducted by comparing the patients discharged to home and a non-home facility for each place of residence. The factors that showed significant differences (*p* < 0.05) in the univariate analysis were included as independent variables in the subsequent stepwise multivariate analysis. A multivariate analysis was performed using logistic regression to identify significant predictors of discharge to home for each place of residence. The possibility of multicollinearity of the independent variables in the multivariate regression analysis was assessed by calculating the variance inflation factor. The FIM adopted only the motor subunit as independent variables in multivariate analysis. Individual (age, sex, and BMI) and social variables (family structure) were selected as covariables. The family structure was based on patients living alone as a reference; patients living as elderly couples or with families were converted into dummy variables and analyzed. A receiver-operating characteristic (ROC) analysis was used to calculate the area under the curve (AUC) and the cutoff point to better distinguish the discharge destination (home or other) by each place of residence. The significance level was set at *p* < 0.05.

## 3. Results

### 3.1. Demographics of the Participants

The demographic characteristics of all the patients are presented in Table 1. The mean ± SD age of all the patients was 84.2 ± 7.0 years, and 392 patients (89.7%) were female. On the basis of their place of residence before the fracture, 437 patients were classified into three groups as follows: 193 patients living in flatter neighborhoods (44.2%); 182, in sloped neighborhoods (41.6%); and 62, in islands (14.2%). Overall, 141 patients (75.5%) in the flat group, 137 (75.3%) in the slope group, and 53 (85.5%) in the island group were discharged to their own homes. The rate of discharge to home was not significantly different among the groups, and no significant differences were found in any other demographic characteristics among the groups.

### 3.2. Comparison of Functional Outcomes Among the Groups

Table 2 depicts the variables for cognitive functions, physical functions, and ADL ability for each group among the discharge destinations. A significant interaction between the group and discharge destination was detected for HDS-R (*f* = 3.10, *p* < 0.05) and 6MWD (*f* = 3.03, *p* < 0.05). All three groups demonstrated higher HDS-R scores among the discharge destinations (*p* < 0.01), and patients of the slope group who were discharged to their homes showed higher HDS-R scores than patients of the flat and island groups (*p* < 0.05) who were discharged to their homes. The 6MWD test also showed higher scores in all three groups among the discharge destinations (*p* < 0.01), and walking endurance of slope group was higher than that of flat and island group (*p* < 0.05).

### 3.3. Functional Predictors and Cutoff Values for Home Discharge

The results of the univariate and multivariate logistic regression analyses for identifying significant predictors of discharge to home for each place of residence are shown in Tables 3, 5 and 6, and the results of the ROC analysis are shown in Table 4.

In the flat group, the univariate analysis revealed significant differences in all the variables: namely HDS-R, grip strength, 10mWT score, 6MWD, OLST, and FIM-motor, cognitive function, and total scores (*p* < 0.01; Table 3). The crude model of the multivariate logistic regression analysis revealed a significant association between discharge to home and FIM-motor scores (odds ratio [OR], 1.10; 95% confidence interval [CI], 1.05–1.16; *p* < 0.01; Table 3). After adjustment for individual and social covariates (adjusted model), the same trend remained. No significant association was observed between discharge to home and any other cognitive or physical function. The FIM-motor score with a cutoff point of 69 gave the best tradeoff with 71% sensitivity and 85% specificity and an AUC of 0.84 (95% CI, 0.76–0.90; Table 4).

Table 5 presents the results of the univariate and multivariate logistic regression analyses for the slope group. The univariate analysis revealed significant differences in all the variables, namely HDS-R score, grip strength, 10mWT score, 6MWD, OLST, and FIM-motor, cognitive function, and total scores (*p* < 0.01). In the crude model of the multivariate analysis, 6MWD (OR, 1.02; 95% CI, 1.01–1.04; *p* < 0.05) and FIM-motor (OR, 1.06; 95% CI, 1.01–1.12; *p* < 0.05) were identified as methods for determining the discharge destination. After adjustment for the individual and social covariates (adjusted mode), HDS-R score was also detected as a predictor of discharge to home (OR, 1.12; 95% CI, 1.10–1.24; *p* < 0.05). The cutoff values for the HDS-R score, 6MWD, and FIM-motor score were 18 points (sensitivity: 84%, specificity: 82%; AUC, 0.85; 95% CI, 0.76–0.92), 150 m (sensitivity: 83%, specificity: 74%; AUC, 0.86; 95% CI, 0.78–0.91), and 65 points (sensitivity: 79%, specificity: 88%; AUC, 0.88; 95% CI, 0.80–0.93), respectively (Table 4).

Table 6 presents the results of the univariate and multivariate logistic regression analyses for the island group. The univariate analysis revealed significant differences in HDS-R, 6MWD, and FIM-total scores (*p* < 0.05). Similar to the flat group, the crude and adjusted models of the multivariate analysis showed a significant association between discharge to home and FIM-motor scores (OR, 1.17; 95% CI, 1.03–1.33; *p* < 0.05 and OR, 1.21; 95% CI, 1.04–1.42; *p* < 0.05, respectively). No significant association was observed between discharge to home and any other cognitive and physical functions in the crude and adjusted models. The cutoff FIM-motor score was 65, with 71% sensitivity, 85% specificity, and an AUC of 0.84 (95% CI, 0.58–0.88; Table 4).

## 4. Discussion

This is the first study to reveal that the predictors of discharge to home of patients after a hip fracture differ depending on the neighborhood terrain, especially for patients living in areas with many slopes and stairs.

The results of the logistic regression analysis indicated that the FIM-motor score was a significant predictor of discharge to home for the patients in all the groups. A previous study showed that FIM-motor score may be an effective indicator for predicting and determining whether patients with hip fracture could be discharged to their homes [19,20]. Wang et al. [20] discriminated that the 58 points of the FIM-motor is an important indicator that distinguishes discharge to home or other facilities. The cutoff value with high sensitivity and specificity was 69 points for the flat group and 65 points for the slope and island groups in our study. The 65 and 69 points of the FIM-motor represent the range between the mean ratings of “supervision: 5 points” and “modified independence: 6 points”’ across the 13 items. Although some studies reported not having a spouse or other relatives is a determining factor of failure of home discharge [14], the same trend remained for the result of the analysis of the relationship between FIM-motor score and home discharge in our study after adjustment for family structure. Therefore, the present findings indicate that patients performing ADL at or above almost the “supervision” level 4–5 weeks after their admission, who can live almost independently on their own, are more likely to be discharged to their homes regardless of the neighborhood terrain and family structure.

For patients living in sloped residential areas, FIM-motor score, 6MWD, and HDS-R score were identified as predictors of discharge to home, and 6MWD was a significant predictor specific to the slope group independent of individual and social covariates. Elderly people living in sloped areas are less likely to walk than those living in flatter areas [21], and the sloped terrain may lead to barriers for walking [8]. In Kure City, many elderly people live in areas where the altitude difference between the bus stop and their home is >50 m, and they need to walk at least 200 m on slopes and stairs to the bus stop [15]. Furthermore, many residential areas have slope angles of 30–40 ° and road widths of 70 m with no handrails [15]. For patients living in areas with many slopes and stairs, walking durability is an essential factor for discharge to home on a hillside terrain, including Kure City, and they need to regain the ability to walk to the point of being able to walk continuously for at least 150 m 4–5 weeks after their admission.

Patients with cognitive deficits have a low probability of returning to their own home [11,12,22]. From the adjusted logistic regression model by individual and social covariates in this study, a significant association between discharge to home and HDS-R score emerged. Increasing age and cognitive deficit were risk factors that affected the home discharge of patients after hip fracture [22]. The cutoff HDS-R value for determining the presence or absence of dementia is 20 points [16], and the optimal cutoff value of 18 points calculated in this study corresponds to mild dementia. Takeda et al. reported that the reduction in FIM score in ADL after hip fracture is largely attributable to cognitive function [19]. On the basis of these facts, in the post-hip fracture rehabilitation of patients living in areas with slope or stairs, whether the patient meets the HDS-R criteria for home discharge 4–5 weeks after their admission must be determined, considering the patient’s age and family structure, and rehabilitation should focus on preparation for home discharge, including more stairs/slope training for the slope group.

The results of the two-way ANOVA showed that HDS-R and 6MWD were higher for patients of home discharge in the sloping group compared to the other groups. A study in the United States found that the presence of slopes increased the odds of physical activity by 26% [23], and cognitive disorder did not progress during the short period after hip fracture [19]. Therefore, we interpreted as the high cognitive function and walking endurance of home discharged patients in the slope group were reflected their preoperative abilities. Cognitive impairment predicts poor postoperative functional recovery [24]. Although it has been reported that the rate of regaining preoperative walking ability was 40–57% [4,5], postoperative rehabilitation is particularly important in the slope group, since incline group requires a higher level of walking ability, as mentioned above. In the slope group, patients with relatively higher cognitive function may have benefited from postoperative rehabilitation, and high cognitive function may have facilitated the recovery of walking ability after surgery. On the other hand, patients with lower cognitive function before the surgery who managed to live with family support may have been affected by cognitive impairment during their postoperative rehabilitation and had to be discharged to care facilities due to their walking ability did not recover to the preoperative level.

The population of Kure City has been aging remarkably, and >60% of the patients in this study were living alone or living as elderly couples, however, the percentage of patients returning to their own homes was between 73% and 86%, regardless of the place of residence before the fracture. The rate of discharge to home from convalescent units was 78% [25], which is similar to the current results. Most patients who returned to their homes within 1 year after a hip fracture were still able to live in their homes 10 years later [2,3]. Therefore, it is important to discharge as many patients as possible to their homes to extend their healthy life expectancy and quality of life. With earlier consideration of the discharge destination 4–5 weeks after their admission by using the criteria presented in this study, the patients who did not meet the criteria may be able to return home with a comprehensive management of the remaining hospitalization period, including adjustment of their home environment and considering in-home care support.

The major problem with hip fracture is that even a single hip fracture can cause loss of functional status and a higher risk for a second hip fracture [26]. The patients in this study were discharged approximately 59 days (8 weeks) after admission to the convalescent unit; however, even for those who were discharged to home, their walking speeds and balance ability were lower than the reference values for assessing the risk of falls [27,28]. Functional recovery can be expected for at least 6 months after surgery for hip fracture [2], and the effectiveness of training at home has been represented [29]. To prevent secondary fractures, continuous follow-up after discharge was required.

This study has some limitations. First, factors, including economic status, social support, and severity of comorbidities, that we did not investigate might be associated with discharge destination. Second, to assure the accuracy of the group dividing, we took sufficient precautions, such as checking the neighborhood terrain with maps, however, it was difficult to calculate specific values for the determining the differences in the degree of slope of each group objectively. Third, the limited sample size of the patients living in islands might have provided insufficient data to examine multiple outcomes and interactions among the predictors. Therefore, large follow-up studies of people living in islands must be conducted to ascertain factors and cutoff scores that best differentiate patients discharged to their homes.

## 5. Conclusions

The FIM-motor score showed good discriminative ability to predict discharge destination of patients after hip fracture regardless of the geographical features surrounding their homes, and a score of 65 or 69 points was shown to be useful for classifying patients discharged to their homes and those admitted to institutions. Furthermore, the walking endurance and cognitive functions of patients being discharged to their homes located in areas with many slopes and stairs must be considered. The study results may be useful for determining discharge plans and setting rehabilitation goals.

## Figures and Tables

**Table 1:** Demographic characteristics of the subjects in the three groups.

Variables	Total (*n* = 437)	Flat Group (*n* = 193)	Slope Group (*n* = 182)	Island Group (*n* = 62)	*p*-value
Sex					
Male Female	45 (10.3) 392 (89.7)	21 (10.7) 172 (89.1)	16 (8.8) 166 (91.2)	8 (12.9) 54 (87.1)	0.85
Age (years)	84.2 ± 7.0	84.9 ± 6.7	84.0 ± 7.3	82.5 ± 6.9	0.06
Height (cm)	148.3 ± 8.0	148.2 ± 8.5	148.4 ± 7.9	148.0 ± 6.9	0.92
Weight (kg)	45.9 ± 9.3	45.1 ± 9.2	46.5 ± 9.9	46.7 ± 7.5	0.26
BMI (kg/m^2^)	20.8 ± 3.4	20.4 ± 3.4	21.0 ± 3.6	21.2 ± 2.7	0.15
Fracture type					
Femoral neck Trochanteric	188 (43.0) 249 (57.0)	85 (44.0) 108 (56.0)	76 (41.8) 106 (58.2)	27 (43.5) 35 (56.5)	0.97
Operative procedure					
Osteosynthesis Arthroplasty	269 (61.6) 168 (38.4)	116 (60.1) 77 (39.9)	113 (62.1) 69 (37.9)	40 (64.5) 22 (35.5)	0.60
Family structure					
Alon Elderly couple With family	166 (38.0) 105 (24.0) 166 (38.0)	78 (40.4) 50 (25.9) 65 (33.7)	65 (35.7) 39 (21.4) 78 (42.9)	23 (37.1) 16 (25.8) 23 (37.1)	0.33
Discharge destination					
Own home Other	331 (75.7) 106 (24.3)	141 (73.1) 52 (26.9)	137 (75.3) 45 (24.7)	53 (85.5) 9 (14.5)	0.13
Hospitalization (days)	59.0 ± 20.8	59.4 ± 20.4	59.5 ± 21.1	56.2 ± 21.4	0.54

Values are presented as mean ± standard deviation or *n* (%). BMI = body mass index.

**Table 2:** Results of two-way ANOVA for comparison of cognitive/physical and ADL outcomes.

Variables	Flat Group (*n* = 193)		Slope Group (*n* = 182)		Island Group (*n* = 62)		Interaction Effect		Main Effect (Group)		Main Effect (Discharge Destination)	
	Own Home (*n* = 141)	Other (*n* = 52)	Own Home (*n* = 137)	Other (*n* = 45)	Own Home (*n* = 53)	Other (*n* = 9)	*f*-Value	*p*-Value	*f*-Value	*p*-Value	*f*-Value	*p*-Value
HDS-R (points)	21.2 ± 6.9	14.4 ± 8.2	23.2 ± 6.1 ^a, b^	12.6 ± 7.6	19.0 ± 6.9	14.1 ± 7.8	3.10	<0.05	0.37	0.69	45.4	<0.01
Grip strength (kg)	12.9 ± 5.4	9.0 ± 4.0	13.7 ± 5.9	9.1 ± 4.0	12.0 ± 3.9	12.0 ± 2.7	1.69	0.19	0.66	0.52	9.53	<0.01
10mWT (seconds)	15.3 ± 9.3	19.4 ± 10.2	13.1 ± 6.2	19.9 ± 9.7	16.1 ± 9.6	20.2 ± 14.1	0.84	0.43	0.50	0.61	12.9	<0.01
6MWD (meter)	203.6 ± 93.2	142.6 ± 54.8	232.0 ± 91.1 ^a, b^	132.4 ± 53.6	199.5 ± 94.5	139.5 ± 68.4	3.03	<0.05	0.04	0.96	21.8	<0.01
OLST (seconds)	5.2 ± 11.5	0.8 ± 0.8	4.9 ± 8.5	1.2 ± 2.1	4.0 ± 7.6	0.8 ± 1.2	0.08	0.92	0.06	0.94	7.26	<0.01
FIM (points)												
Motor	74.5 ± 12.1	54.2 ± 17.1	75.4 ± 12.0	53.5 ± 15.2	69.4 ± 14.2	51.9 ± 11.4	0.33	0.72	1.01	0.37	94.7	<0.01
Cognitive	28.6 ± 6.2	20.2 ± 8.7	29.5 ± 6.4	19.1 ± 6.6	26.8 ± 6.9	22.3 ± 6.9	2.27	0.11	0.02	0.98	56.7	<0.01
Total	103.1 ± 17.1	74.4 ± 24.6	104.9 ± 16.9	72.6 ± 20.3	96.2 ± 18.8	74.1 ± 14.7	0.91	0.40	0.46	0.63	94.2	<0.01

Values are presented as the mean ± standard deviation. HDS-R = revised Hasegawa dementia scale; 10mWT = 10-m walking time; 6MWD = 6-min walking distance; OLST = one-leg standing time; FIM = functional independence measure. Values are presented as mean (standard deviation). a: Significant difference between patients discharged to own home in slope group and in flat group (*p* < 0.05). b: Significant difference between patients discharged to own home in slope group and in island group (*p* < 0.05).

**Table 3:** The cognitive/physical and ADL outcomes in the flat group and the functional predictors for home discharge.

Variables	Univariate Analysis		Multivariate Analysis			
			Crude Model		Adjusted Model	
	OR (95% CI)	*p*-Value	OR (95% CI)	*p*-Value	OR (95% CI)	*p*-Value
HDS-R	1.12 (1.07–1.18)	<0.01	1.01 (0.94–1.09)	0.73	1.01 (0.93–1.08)	0.86
Grip strength	1.22 (1.11–1.36)	<0.01	1.05 (0.95–1.19)	0.36	1.06 (0.93–1.21)	0.36
10mWT	0.96 (0.93–0.99)	<0.05	1.01 (0.95–1.08)	0.69	1.01 (0.94–1.08)	0.89
6MWD	1.01 (1.00–1.01)	<0.01	1.00 (0.99–1.01)	0.65	1.00 (0.99–1.01)	0.56
OLST	1.78 (1.26–2.86)	<0.01	1.31 (0.98–2.25)	0.25	1.22 (0.76–1.96)	0.41
FIM						
Motor	1.09 (1.06–1.13)	<0.01	1.10 (1.05–1.16)	<0.01	1.11 (1.05–1.17)	<0.01
Cognitive	1.16 (1.10–1.22)	<0.01	−	–	–	–
Total	1.02 (1.01–1.02)	<0.01	−	−	–	–

ADL = activities of daily living; HDS-R = revised Hasegawa dementia scale; 10mWT = 10-m walking time; 6MWD = 6-min walking distance; OLST = one-leg standing time; FIM = functional independence measure; OR = odds ratio; CI = confidence interval. The adjusted model of the multivariate analysis was adjusted for individual (age, sex, and BMI) and social (family structure) covariates. Variance inflation factor: HDS-R, 1.34; grip strength, 1.14; 10mWT, 1.54; 6MWD, 1.91; OLST, 1.32; FIM-motor, 1.61.

**Table 4:** The cutoff value required for discharge to home for each place of residence.

	Cutoff	Sensitivity	Specificity	AUC (95% CI)
Flat group				
FIM-motor	69 points	71%	85%	0.84 (0.76–0.90)
Slope group				
HDS-R	18 points	84%	82%	0.85 (0.76–0.92)
6MWD	150 m	83%	74%	0.86 (0.78–0.91)
FIM-motor	65 points	79%	88%	0.88 (0.80–0.93)
Island group				
FIM-motor	65 points	71%	88%	0.76 (0.58–0.88)

HDS-R = revised Hasegawa dementia scale; 6MWD = 6-min walking distance; FIM = functional independence measure; AUC = area under the curve; CI, confidence interval.

**Table 5:** Cognitive/physical and ADL outcomes in the slope group and functional predictors for home discharge.

Variables	Univariate Analysis		Multivariate Analysis			
			Crude Model		Adjusted Model	
	OR (95% CI)	*p*-Value	OR (95% CI)	*p*-Value	OR (95% CI)	*p*-Value
HDS-R	1.21 (1.14–1.30)	<0.01	1.08 (0.99–1.82)	0.08	1.12 (1.10–1.24)	<0.05
Grip strength	1.22 (1.11–1.34)	<0.01	1.01 (0.88–1.17)	0.88	1.01 (0.83–1.22)	0.92
10mWT	0.88 (0.83–0.93)	<0.01	1.01 (0.92–1.10)	0.77	1.04 (0.93–1.16)	0.49
6MWD	1.03 (1.02–1.04)	<0.01	1.02 (1.01–1.04)	<0.05	1.03 (1.01–1.06)	<0.01
OLST	1.31 (1.04–1.65)	<0.01	0.98 (0.85–1.27)	0.85	0.93 (0.77–1.11)	0.40
FIM						
Motor	1.11 (1.07–1.15)	<0.01	1.06 (1.01–1.12)	<0.05	1.06 (1.01–1.14)	<0.05
Cognitive	1.21 (1.14–1.29)	<0.01	–	–	–	–
Total	1.02 (1.01–1.03)	<0.01	–	–	–	–

ADL = activities of daily living; HDS-R = revised Hasegawa dementia scale; 10mWT = 10-m walking time; 6MWD = 6-min walking distance; OLST = one-leg standing time; FIM = functional independence measure; OR = odds ratio; CI = confidence interval. The adjusted model of the multivariate analysis was adjusted for individual (age, sex, and BMI) and social (family structure) covariates. Variance inflation factor: HDS-R, 1.37; grip strength, 1.23; 10mWT, 1.80; 6MWD, 1.86; OLST, 1.16; FIM-motor, 1.44.

**Table 6:** Cognitive/physical and ADL outcomes in the island group and functional predictors for home discharge.

Variables	Univariate Analysis		Multivariate Analysis			
			Crude Model		Adjusted Model	
	OR (95% CI)	*p*-Value	OR (95% CI)	*p*-Value	OR (95% CI)	*p*-Value
HDS-R	1.14 (1.01–1.29)	<0.05	1.15 (0.98–1.35)	0.09	1.18 (0.94–1.48)	0.15
Grip strength	1.03 (0.85–1.24)	0.78				
10mWT	0.96 (0.90–1.02)	0.16				
6MWD	1.01 (1.00–1.02)	<0.05	0.99 (0.97–1.01)	0.42	0.99 (0.97–1.01)	0.32
OLST	1.28 (0.82–2.00)	0.11				
FIM						
Motor	1.09 (1.02–1.16)	<0.01	1.17 (1.03–1.33)	<0.05	1.21 (1.04–1.42)	<0.05
Cognitive	1.11 (0.99–1.24)	0.06	–	–	–	–
Total	1.01 (0.99–1.03)	0.55	–	–	–	–

ADL = activities of daily living; HDS-R = revised Hasegawa dementia scale; 10mWT = 10-m walking time; 6MWD = 6-minute walking distance; OLST = one-leg standing time; FIM = functional independence measure; OR = odds ratio; CI = confidence interval. The adjusted model of the multivariate analysis was adjusted for individual (age, sex, and BMI) and social (family structure) covariates. Variance inflation factor: HDS-R, 1.05; 6MWD, 1.93; FIM-motor, 1.89.
